# The risk of viral rebound in the year after delivery in women remaining on antiretroviral therapy

**DOI:** 10.1097/QAD.0000000000000826

**Published:** 2015-11-04

**Authors:** Susie Huntington, Claire Thorne, Marie-Louise Newell, Jane Anderson, Graham P. Taylor, Deenan Pillay, Teresa Hill, Pat A. Tookey, Caroline Sabin

**Affiliations:** a Population, Policy and Practice Programme, UCL Institute of Child Health; b HIV Epidemiology and Biostatistics Group, UCL Research Department of Infection and Population Health, London; c Human Development and Health, Faculty of Medicine, Southampton General Hospital, University of Southampton, Southampton; d Homerton University Hospital NHS Foundation Trust; e Section of Retrovirology and GU Medicine, Imperial College, London, UK; f Africa Centre for Health and Population Studies, University of KwaZulu-Natal, Mtubatuba, South Africa.

**Keywords:** adherence, HAART, HIV, postpartum women, pregnant women, viral load

## Abstract

The objective of this study is to assess the risk of viral rebound in postpartum women on suppressive combination antiretroviral therapy (cART).

**Methods:**

Using data from the UK Collaborative HIV Cohort (UK CHIC) study and the UK and Ireland National Study of HIV in Pregnancy and Childhood (NSHPC), women with HIV-RNA 50 copies/ml or less at delivery in 2006–2011, who started life-long cART during pregnancy (*n* = 321) or conceived on cART (*n* = 618), were matched by age, duration on cART and time period, with at least one control (non-postpartum). The cumulative probability of viral rebound (HIV-RNA >200 copies/ml) was assessed by Kaplan–Meier analysis; adjusted hazard ratios (aHRs) for the 0–3 and 3–12 months postdelivery (cases)/pseudo-delivery (controls) were calculated in Cox proportional hazards models.

**Results:**

In postpartum women who conceived on cART, 5.9% [95% confidence interval (95% CI) 4.0–7.7] experienced viral rebound by 3 months, and 2.2% (1.4–3.0%) of their controls. The risk of viral rebound was higher in postpartum women than in controls during the first 3 months [aHR 2.63 (1.58–4.39)] but not during the 3–12 months postdelivery/pseudo-delivery. In postpartum women who started cART during pregnancy, 27% (22–32%) experienced viral rebound by 3 months, and 3.0% (1.6–4.4%) of their controls. The risk of viral rebound was higher in postpartum women than in controls during both postdelivery/pseudo-delivery periods [<3 months: aHR 6.63 (3.58–12.29); 3–12 months: aHR 4.05 (2.03–8.09)].

**Conclusion:**

In women on suppressive cART, the risk of viral rebound is increased following delivery, especially in the first 3 months, which may be related to reduced adherence, indicating the need for additional adherence support for postpartum women.

## Introduction

Historically, women living with HIV not yet eligible for life-long combination antiretroviral therapy (cART) used short-course cART or zidovudine monotherapy in pregnancy to prevent mother-to-child transmission, before initiating life-long treatment when their CD4^+^ cell count reached a specified level. However, with expanding CD4^+^ criteria for treatment initiation, the number of women already on life-long cART at conception or who are eligible to start when diagnosed during pregnancy has increased [1–3]. Since 2013, the WHO has recommended that in low and middle-income countries, all pregnant women not yet on treatment start life-long cART [4]. However, in the UK, the use of short-course cART in pregnancy remains an option for women with a CD4^+^ cell count above 350 cells/μl. Pregnant women with a CD4^+^ cell count of 350–500 cells/μl have the option of continuing cART use if there are no contraindications such as poor adherence, as are women with a CD4^+^ cell count more than 500 cells/μl with a discordant partner [5]. Thus, an increasing proportion of women now remain on cART after pregnancy.

Viral rebound generally occurs rapidly following cessation of short-course cART after delivery [6–9]. However, viral rebound has also been observed in postpartum women remaining on cART, even when viral suppression was achieved in pregnancy [9–11]. Using pooled data from two observational studies, we assess, among women on suppressive cART, the risk of viral rebound in women with a pregnancy in the previous year and in matched controls who had not been pregnant, with the postpartum group stratified by timing of cART initiation (before or during pregnancy).

## Materials and methods

### Data collection

The UK Collaborative HIV Cohort (UK CHIC) study is an ongoing observational study of adults attending HIV clinical care, which annually collates pseudonymised data from (currently 19) UK-based HIV clinics. Data include all viral load measurements, CD4^+^ cell counts, hepatitis B virus (HBV)/hepatitis C virus (HCV) coinfection status, ART drug regimen and demographic information. The UK and Ireland's National Study of HIV in Pregnancy and Childhood (NSHPC) is a comprehensive, observational, active surveillance study of HIV-positive women accessing antenatal care, with data reported by all maternity units in the UK and Ireland [12], including ethnicity, age and expected delivery date, details of ART use, CD4^+^ cell counts and viral loads in pregnancy. Both studies had ethics approval and informed consent was not required.

Record linkage between these two pseudonymised datasets is based on an algorithm that utilizes basic demographic and clinical data. Since 2010, linkage was undertaken yearly using the most recent datasets [13].

A woman was categorized as having attended for clinical care if any viral load or CD4^+^ cell count data were reported to UK CHIC during the period of interest. ART use at conception, delivery and within 6 months of delivery was assessed using data from both studies.

Women with a pregnancy resulting in a live birth in 2006–2011, an HIV-RNA 50 copies/ml or less at latest viral load 3 months or less before delivery and who remained on cART (use of at least three ART drugs) for at least 6 months after delivery and with at least one viral load measurement in the year after delivery were included in this analysis, including only a woman's earliest pregnancy meeting the criteria.

Two controls were sought from the UK CHIC dataset for each postpartum woman. Controls were HIV-positive women accessing HIV-related care who had not recently been pregnant. For women who had conceived on cART, controls were matched on age (by year), calendar year and number of years since starting life-long ART. For women who started life-long cART in pregnancy, controls were matched on the basis of age (grouped as 16–19; 20–24; 25–29; 30–34; 35–39; 40–44; 45–49 years), calendar year (grouped as 2006–2007; 2008–2009; 2010–2011), months since starting treatment (grouped as 0 to <3; 3 to <6; 6 to <9 months) and CD4^+^ cell count when starting treatment (grouped as ≤200; 201–350; 351–500; >500 cells/μl).

To select suitable controls, reference dates were created by splitting the period 2006–2011 into equal-sized intervals and establishing each woman's clinical characteristics (of interest) on each date. The period was split into 3-month intervals, to find controls for women conceiving on cART, and 1-month intervals, for women starting cART in pregnancy. For controls, the reference date was used as the pseudo-delivery date. Eligibility criteria for controls were: not currently pregnant, not pregnant within the previous year, latest viral load 50 copies/ml or less, at least one viral load measurement in the following year and on cART for at least the following 6 months. If multiple potential controls were identified, two were selected at random. For postpartum women conceiving on cART, women could act as controls on multiple occasions for non-overlapping time periods.

The primary outcome was viral rebound (defined as a single measure of HIV-RNA >200 copies/ml) within 12 months of delivery (postpartum women) or pseudo-delivery (controls). In sensitivity analysis, viral rebound was defined as a single measure of HIV-RNA more than 1000 copies/ml.

### Analysis

Characteristics of postpartum women and controls were compared using the chi-square test for categorical variables and Kruskal–Wallis test for continuous (nonnormally distributed) variables. Kaplan–Meier analysis was used to assess the cumulative probability of viral rebound and Cox proportional hazards models to calculate crude and adjusted hazard ratios (aHRs). As the Kaplan–Meier analyses suggested that hazards were likely to diverge after 3 months, separate models are presented for the periods less than 3 and 3–12 months postdelivery/pseudo-delivery, with the latter model including only women who had not experienced viral rebound or censoring during the less than 3-month period. In unadjusted analyses, the baseline characteristics assessed were postpartum status (postpartum/control), CD4^+^ cell count category, type and duration of ART regimen, parity (the number of live births since HIV diagnosis), HBV/HCV coinfection, ethnicity and exposure group. Follow-up was censored at 12 months postdelivery/pseudo-delivery, if a woman died, interrupted ART or became pregnant again, whichever occurred first. In sensitivity analysis, follow-up was also censored if the ART regimen was altered in any way.

## Results

### Postpartum women conceiving on combination antiretroviral therapy and controls

There were 623 postpartum women who conceived on cART, with two controls identified for 607, only one for 11 and none for five women, giving a total of 1225 controls.

The postpartum women and controls were similar with regard to age, year and duration on cART (the matching characteristics) (Table 1). They were also similar with regard to time since HIV-diagnosis (median 5.9 years), type of regimen used [overall, 55% used a nonnucleoside reverse transcriptase inhibitor (NNRTI)-based regimen], the percentage with HBV/HCV coinfection (7% overall) and ethnic group (73% black-African overall). The two groups differed with regard to HIV exposure category, parity, latest CD4^+^ cell count and use of efavirenz (EFV).

In the month following delivery/pseudo-delivery, 10% (64/618) of postpartum women had a viral load measurement and 26% (320/1225) of controls (*P* < 0.001). After 3 months, 70% (435/618) of postpartum women and 70% (862/1225) of controls had had at least one viral load measurement (*P* = 0.99). The median number of viral load measurements overall was 3 [interquartile range (IQR) 2–4] for both groups (*P* = 0.11).

### Viral rebound in postpartum women conceiving on combination antiretroviral therapy and controls

**Fig. 1:**
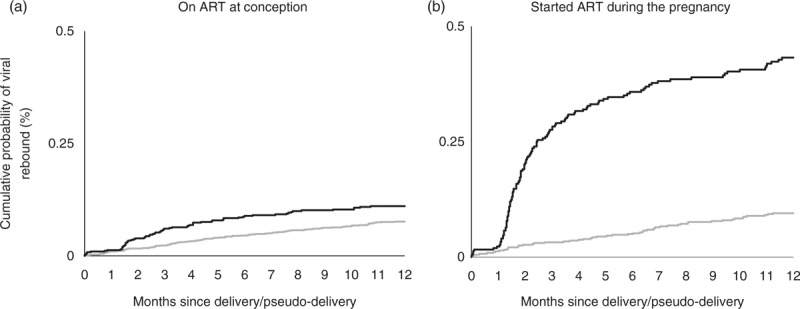
Kaplan–Meier plot showing cumulative probability of viral rebound among women on antiretroviral therapy: postpartum women (black line) and controls (grey line).

A larger percentage of postpartum than control women experienced viral rebound [postpartum: 10.7% (66/618); controls: 7.4% (91/1225)]. The cumulative probability of viral rebound at 1, 3 and 6 months postdelivery/pseudo-delivery was 1.1% [95% confidence interval (95% CI) 0.3–2.0], 5.9% (95% CI 4.0–7.7) and 8.6% (95% CI 6.3–10.8), respectively, in postpartum women, and 0.9 (95% CI 0.0–1.4), 2.2% (95% CI 1.4–3.0) and 4.5% (95% CI 3.3–5.6) in controls (Fig. 1a).

In adjusted analysis, the risk of viral rebound in the 0–3 months postdelivery/pseudo-delivery was associated with postpartum status, calendar year and CD4^+^ cell count (Table 2). Postpartum women were more likely to experience viral rebound than controls (aHR 2.63), although the risk of viral rebound itself decreased in later calendar years (aHR 0.81 per later year). A CD4^+^ cell count of 200 cells/μl or less at delivery/pseudo-delivery was also significantly associated with viral rebound (aHR 2.89).

The risk of viral rebound in the 3–12 months postdelivery/pseudo-delivery was associated with years since HIV diagnosis, type of drug regimen and number of drugs. In this subgroup, who had maintained viral suppression for at least 3 months, there was no statistically significant association between viral rebound and postpartum status. Women who were diagnosed with HIV more than 10 years ago were more likely to experience viral rebound than women diagnosed 2–10 years ago (aHR 1.83). Women on a drug regimen containing at least four drugs were more likely to experience viral rebound than women on a triple regimen (aHR 2.41) as were women on a protease inhibitor-based regimen compared with women on a NNRTI-based regimen (aHR 1.89). The use of EFV was not associated with viral rebound and was not included in the model.

### Postpartum women starting combination antiretroviral therapy in pregnancy and controls

There were 363 postpartum women who started cART during pregnancy, with two controls identified for 247, one for 74 and none for 42 women, giving a total of 568 controls.

Postpartum women and controls were similar with regard to age, year, duration on cART and CD4^+^ cell count when starting cART (the matching characteristics) (Table 1). They were also similar with regard to the type of drug regimen used but differed with regard to ethnicity, exposure category, parity, HBV/HCV coinfection, duration since HIV diagnosis and latest CD4^+^ cell count. On average, postpartum women had been diagnosed more recently, had a higher median CD4^+^ cell count (391 vs. 350 cells/μl), were less likely to use EFV and were less likely to have had a previous live birth (since HIV diagnosis) (Table 1).

More than half [53% (171/321)] of postpartum women were diagnosed with HIV during the recent pregnancy, of whom 65% (111/171) had a CD4^+^ cell count less than 350 cells/μl when starting cART, with 47% (81/171) having a CD4^+^ cell count less than 200 cells/μl. In women already diagnosed when they became pregnant, 59% (89/150) had a CD4^+^ cell count less than 350 cells/μl when starting cART, with 32% (48/150) having a CD4^+^ cell count less than 200 cells/μl; of these, almost two-thirds [65% (98/150)] attended care at a UK CHIC site in the year prior to the pregnancy, of whom 53% (52/98) started cART with a CD4^+^ cell count less than 350 cells/μl.

In the month following delivery/pseudo-delivery, 14% (44/321) of postpartum and 27% (152/568) of controls (*P* < 0.001) had a viral load measurement. At 3 months postdelivery/pseudo-delivery, 80% (256/321) of postpartum women and 79% (450/568) of controls had had at least one viral load measurement (*P* = 0.85). The median number of viral load measurements in the year following delivery/pseudo-delivery was 3 (IQR 2–4) for the postpartum women and 3 (3–4) for the controls (*P* < 0.001).

### Viral rebound in postpartum women starting combination antiretroviral therapy in pregnancy and controls

A larger percentage of postpartum women experienced viral rebound than controls [postpartum: 37.1% (119/321); controls: 9.2% (52/568)]. The cumulative probability of viral rebound at 1, 3 and 6 months postdelivery/pseudo-delivery was 1.9% (95% CI 0.4–3.5), 27% (95% CI 22–32) and 35% (95% CI 30–41), respectively, in postpartum women, and 1.1% (95% CI 0–1.9), 3.0% (95% CI 1.6–4.4) and 4.8% (95% CI 3.0–6.6), respectively, in controls (Fig. 1b).

In adjusted analysis, the risk of viral rebound in the first 3 months postdelivery/pseudo-delivery was associated with postpartum status (aHR 6.63 for postpartum women) and CD4^+^ cell count (aHR 0.18; CD4^+^ cell count <200 vs. >500 cells/μl) (Table 3).

The risk of viral rebound in the 3–12 months postdelivery/pseudo-delivery was associated with postpartum status (aHR 4.05 for postpartum women), calendar year (aHR 0.83 per later year), age (aHR 0.51 per 10 additional years) and ethnicity (aHR 2.94 for women of black-Caribbean ethnicity) (Table 3).

The findings were not affected when, in sensitivity analysis, follow-up was censored at any regimen change.

In sensitivity analysis, when viral rebound was defined as HIV-RNA more than 1000 copies/ml, similar associations were observed, although some lost statistical significance. The association between age and viral rebound became statistically significant for women who had started cART in pregnancy and controls [0–3 months postdelivery/pseudo-delivery: aHR 0.46 (0.24–0.88) per 10 additional years]. Postpartum status remained associated with viral rebound when 143 women (46 postpartum and 97 controls) with previous cART experience were excluded.

## Discussion

We show that HIV-positive women on cART with a live-born infant in the preceding year and an undetectable viral load at delivery had a higher risk of viral rebound than matched control women who had not recently been pregnant. Among women already on cART at conception, the risk of viral rebound was 2.6-fold higher in the first 3 months after delivery than among matched controls, but similar in the 3–12 months after delivery. In contrast, among women who started cART during pregnancy, viral rebound risk was 6.6-fold higher than matched controls during the first 3 months and 4.1-fold higher 3–12 months after delivery. A number of studies have observed a high prevalence of viral rebound in postpartum women remaining on cART [7,10,14], but this study is the first to compare the risk of viral rebound in postpartum women with rates seen in a demographically matched group of non-postpartum women.

Overall, 9% of women who conceived on cART experienced viral rebound within 6 months of delivery, less than in a Brazilian study in which 15% (nine out of 52) of postpartum women, who conceived on and remained on cART after pregnancy, developed viral rebound (0.5 log_10_ increase) at 6 months postpartum [14]. This difference may be because women in the Brazilian study had more advanced disease and not all had achieved viral suppression during pregnancy. Two further studies [7,10] reported that 19 and 18% (respectively) of postpartum women who remained on cART experienced viral rebound (defined as ≥0.7 log_10_ increase at 24 weeks postpartum and ≥0.5 log_10_ increase at 6–12 weeks postpartum, respectively). However, neither of these studies stratified by timing of cART initiation (before or during the pregnancy), which limits comparison with our study.

Physiological changes during pregnancy and at delivery may result in a temporary viral load peak and may have contributed to the increased incidence in the first 3 months after delivery. However, this temporary peak is most likely to occur shortly after delivery [8], a time during which few women in our study had a viral load measurement. Also, this would not explain the ongoing increased risk of viral rebound after 3 months among women who started cART in pregnancy or the much higher incidence in women who started cART in pregnancy than women conceiving on cART. The more likely explanation for the increase in viral rebound incidence following pregnancy is reduced adherence to cART. Studies have observed a fall in adherence following pregnancy [10,15,16], when the risk of vertical transmission has passed (if breastfeeding is avoided) and the demands of looking after the baby are high. Treatment interruptions and changes to medication are also more likely in this period [10]. When, in sensitivity analysis, follow-up was censored if the regimen was changed, the association between post-pregnancy status and viral rebound remained.

Older age was associated with a decreased risk of viral rebound in women who started ART in pregnancy and their controls. This is likely to be a result of better drug adherence in older women [17–19].

Engagement with HIV care may have also been reduced following pregnancy; in our observational study, data were collected as part of HIV clinical care. We used the average number of viral load measurements recorded as a proxy for clinic attendance. For all groups, the median number of viral loads was three in the postdelivery/pseudo-delivery year. However, for women who recently started cART, there was evidence that postpartum women attended care less often than controls (*P* < 0.001 for the distribution of viral load measurements). Previous studies have noted low attendance rates in clinical care in the 3 months following childbirth [20] and delay in seeking HIV care [21] or inability to complete postpartum follow-up [22] among women with children in the household.

We cannot rule out resistance as the reason for viral rebound; however, in sensitivity analysis excluding women with known previous exposure to ART, postpartum status remained significantly associated with an increased risk of viral rebound.

In women who started cART in pregnancy and their controls, the risk of viral rebound in the first 3 months after delivery/pseudo-delivery was lower in women with a low CD4^+^ cell count than in women with a high CD4^+^ cell count. Despite only including women who, according to the data, remained on cART for at least 6 months, some discontinuations may not have been recorded in the clinical notes. A woman's CD4^+^ cell count at cART initiation could indirectly affect adherence; for example, women with a high CD4^+^ cell count may not perceive the need for perfect adherence as much as women starting cART with a low CD4^+^ cell count. In some settings wherein WHO Option B+ has been implemented, high rates of lost-to-follow-up have been observed among pregnant women starting life-long cART with a high CD4^+^ cell count [23,24]. We do not know whether a similar incidence of postpartum viral rebound would occur if all pregnant women not yet on ART started long-term treatment in pregnancy, as is increasingly the case in low and middle-income settings [4]. Remaining on treatment after pregnancy may be beneficial for the woman's health and to minimize HIV transmission risk in subsequent pregnancies [25,26]. The PROMISE study is currently assessing the benefits, in a resource-limited settings, of women with higher CD4^+^ cell counts remaining on cART after delivery (Trial reference: NCT01061151).

For women who started cART in pregnancy and their controls, a quadruple regimen was associated with an increased risk of viral rebound in the 3–12 months postdelivery/pseudo-delivery. As the standard first-line treatment in the UK during the study period was a triple regimen (wherein ritonavir use as a pharmacological booster is not counted as a component of the regimen) [27], use of a quadruple regimen suggests that they were on a subsequent regimen due to developing resistance or problems with a previous regimen/s. Adherence could be more of an issue for women on a quadruple regimen, as adherence is negatively associated with pill burden [28].

In both groups, fewer postpartum women were on an EFV-containing regimen than the controls. Until recently, EFV has been avoided in pregnancy and in women planning a pregnancy due to the possible risk to foetal development [29], although a recent meta-analysis found no increase in birth defects with EFV use [30]. In women who started ART in pregnancy and their controls, use of EFV was associated with a lower risk of viral rebound in the first 3 months postdelivery/pseudo-delivery. No such association was found in women who had conceived on ART.

Although several relevant variables were included in our adjusted model, we may not have accounted for all potential confounders. To avoid detecting viral blips, viral rebound is often defined on the basis of two consecutive HIV-RNA more than 200/400/1000 copies/ml; we were unable to take this approach due to the limited number of viral load measurements reported in this group.

Increased viral load following pregnancy could have a detrimental impact on women's health and future treatment options and increases the risk of transmission to an HIV-negative partner, or to the infant, if the mother chooses to breastfeed. Therefore, our findings indicate a need for additional support for ART adherence and to remain engaged in regular HIV clinical care, which could include support from clinicians, specialist nurses and peer support (via charities) from women living with HIV who have experience of taking ART after pregnancy. The findings of this study suggest that adherence support is particularly needed by women starting life-long treatment during pregnancy, especially younger women. It is encouraging that the risk of viral rebound was lower in later years of the study, indicating that adherence may have improved over time and that regimens have become more forgiving to lapses in adherence. The UK CHIC study does not collect data on pill burden or use of fixed-dose regimens (FDRs), so these could not be assessed as potential factors associated with viral rebound. However, other studies have found that use of a single-pill regimen can improve adherence [31]. For pregnant women starting long-term cART, a once-a-day FDR may promote good adherence. Regimens more forgiving to poor adherence could also be considered as the initial regimen. Further studies are required to identify the most effective strategies for improving postpartum ART adherence.

In conclusion, in women on suppressive cART, the risk of viral rebound is higher in postpartum women than in similar women who have not recently had a pregnancy. This may be a result of reduced adherence to ART, highlighting the need for additional adherence support for pregnant and postpartum women remaining on cART.

## Acknowledgements

S.H. carried out the statistical analysis and drafted the manuscript. T.H. undertook data acquisition. All coauthors contributed to the interpretation and drafting of the manuscript.

UK CHIC steering committee included: J. Ainsworth, S. Allan, J. Anderson, A. Babiker, D. Chadwick, V. Delpech, D. Dunn, M. Fisher, B. Gazzard (Chair), R. Gilson, M. Gompels, P. Hay, T. Hill, M. Johnson, S. Kegg, C. Leen, F. Martin, M. Nelson, C. Orkin, A. Palfreeman, A. Phillips, D. Pillay, F. Post, J. Pritchard, C. Sabin (PI), A. Schwenk, A. Tariq, R. Trevelion, J. Walsh.

UK CHIC central co-ordination: Research Department of Infection & Population Health, UCL, London (T. Hill, S. Huntington, S. Jose, A. Phillips, C. Sabin, A. Thornton); Medical Research Council Clinical Trials Unit (MRC CTU), London (D. Dunn, A. Glabay).

UK CHIC participating sites: Barts & The London NHS Trust, London (C. Orkin, J. Lynch, J. Hand, C. de Souza); Brighton and Sussex University Hospitals NHS Trust (M. Fisher, N. Perry, S. Tilbury, D. Churchill); Chelsea and Westminster NHS Trust, London (B. Gazzard, M. Nelson, M. Waxman, D. Asboe, S. Mandalia); Public Health England (PHE), Centre for Infections, London (V. Delpech); Homerton University Hospital NHS Trust, London (J. Anderson, S. Munshi, D. Awosika); King's College Hospital, London (F. Post, H. Korat, C. Taylor, Z. Gleisner, F. Ibrahim, L. Campbell); UCL Medical School and The Mortimer Market Centre, London (R. Gilson, N. Brima, I. Williams); North Bristol NHS Trust (M. Gompels, S. Allen); North Middlesex University Hospital NHS Trust, London (A. Schwenk, J. Ainsworth, C. Wood, S. Miller); Royal Free NHS Trust & Department of Infection & Population Health, UCL, London (M. Johnson, M. Youle, F. Lampe, C. Smith, H. Grabowska, C. Chaloner, D. Puradiredja); Imperial College Healthcare NHS Trust, London (J. Walsh, N. Mackie, A. Winston, J. Weber, F. Ramzan); The Lothian University Hospitals NHS Trust, Edinburgh (C. Leen, A. Wilson); University of Leicester NHS Trust (A. Palfreeman, A. Moore, L. Fox); South Tees Hospitals NHS Foundation Trust (D. Chadwick, K. Baillie); Woolwich NHS Trust (S. Kegg, P. Main); Coventry & Warwickshire NHS Trust (S. Allan); St. George's NHS Trust (P. Hay, M. Dhillon); York NHS Foundation Trust (F. Martin, S. Douglas); The Royal Wolverhampton NHS Trust (A. Tariq); Ashford and St Peter's Hospital NHS Foundation Trust (J. Pritchard).

NSHPC Steering Committee included: M. Cortina-Borja, A. Brown, A. de Ruiter, S. Donaghy, S. Farthing, K. Harding, A. Judd, L. Logan, H. Lyall, A. Namiba, F. Ncube, C. Peckham (chair), L. Primrose, C. Thorne, P. Tookey (PI), S. Webb.

We gratefully acknowledge the contribution of the midwives, obstetricians, genitourinary physicians, paediatricians, clinical nurse specialists and all other colleagues who report to the NSHPC through the British Paediatric Surveillance Unit of the Royal College of Paediatrics and Child Health, and the obstetric reporting scheme run under the auspices of the Royal College of Obstetricians and Gynaecologists.

Ethics approval for NSHPC was renewed following review by the London Multi-Centre Research Ethics Committee in 2004 (MREC/04/2/009).

The UK CHIC is funded by the UK Medical Research Council (MRC) (Grant numbers G0000199, G0600337 and G0900274). The NSHPC receives core funding from PHE (grant number GHP/003/013/003). Data are collated at the UCL Institute of Child Health, which receives a proportion of funding from the Department of Health's National Institute for Health Research Biomedical Research Centres funding scheme.

S.H. has a UCL Studentship, funded by the MRC, for postgraduate work.

### Conflicts of interest

All authors report no potential conflicts.

## Figures and Tables

**Table 1:** Baseline characteristics of postpartum women and controls.

		On ART at conception^a^				*P*	Started ART during the pregnancy^b^				*P*
		Postpartum *n* = 618		Controls *n* = 1225			Postpartum *n* = 321		Controls *n* = 568		
Baseline characteristic^c^		*n*	(%)	*n*	(%)		*n*	(%)	*n*	(%)	
Year^d^	2006–2007	206	(33.4)	407	(33.3)	–	114	(35.5)	207	(36.4)	–
	2008–2009	207	(33.6)	413	(33.7)		99	(30.8)	176	(31.0)	
	2010–2011	205	(33.2)	405	(33.1)		108	(33.6)	185	(32.6)	
Age^d^	Median [IQR] years	34	[31–37]	34	[31–37]	–	31	[28–35]	32	[28–35]	–
Ethnicity	Black African	479	(77.5)	882	(72.0)	0.07	251	(78.2)	375	(66.0)	0.002
	White	61	(9.9)	163	(13.3)		25	(7.8)	78	(13.7)	
	Black Caribbean	14	(2.3)	36	(2.9)		14	(4.4)	30	(5.3)	
	Other/NK	64	(10.4)	139	(11.8)		31	(9.7)	85	(15.0)	
Exposure category	Heterosexual sex	604	(97.7)	1140	(93.1)	0.001	309	(96.3)	520	(91.6)	0.01
	Injecting drug use	6	(1.0)	31	(2.5)		0	–	10	(1.8)	
	Other/NK	8	(1.3)	54	(4.4)		12	(3.7)	38	(6.7)	
Parity^e^	0	353	(57.1)	855	(69.8)	<0.001	280	(87.2)	470	(82.8)	<0.01
	1	196	(31.7)	252	(20.6)		37	(11.5)	68	(12.0)	
	≥2	69	(11.2)	118	(9.6)		4	(1.3)	30	(5.3)	
HBV/HCV coinfection		37	(6.0)	93	(7.6)	0.20	8	(2.5)	46	(8.1)	0.001
Latest CD4^+^ cell count (cells/μl)	≤200	39	(6.3)	58	(4.7)	<0.001	51	(15.9)	113	(19.9)	0.05
	201–350	153	(24.8)	201	(16.4)		93	(29.0)	172	(30.3)	
	351–500	191	(31.0)	315	(25.7)		81	(25.2)	159	(28.0)	
	>500	234	(37.9)	651	(53.1)		96	(29.9)	124	(21.8)	
Median time since HIV diagnosis [IQR] (years)		5.9	[3.7–8.3]	5.9	[3.6–8.7]	0.33	0.6	[0.5–3.8]	2.8	[0.7–6.5]	<0.001
Duration of current period of ART use^d^	0–2 months	–	–	–	–	–	65	(20.3)	106	(18.7)	–
	3–5 months	–	–	–	–		233	(72.8)	416	(73.2)	
	6–8 months	–	–	–	–		22	(6.9)	46	(8.1)	
	8–12 months	39	(6.3)	78	(6.4)		–	–	–	–	
	1–4 years	394	(63.8)	785	(64.1)		–	–	–	–	
	≥5 years	185	(29.9)	362	(29.6)		–	–	–	–	
Type of ART regimen	PI	221	(35.8)	404	(33.0)	0.49	84	(26.2)	160	(28.2)	0.68
	NRTI	7	(1.1)	22	(1.8)		3	(0.9)	3	(0.5)	
	NNRTI	332	(53.7)	676	(55.2)		221	(68.9)	388	(68.3)	
	Other	58	(9.4)	123	(10.0)		13	(4.1)	17	(3.0)	
EFV-containing regimen		88	(14.2)	407	(33.2)	<0.001	11	(3.4)	293	(51.6)	<0.001
Number of drugs in regimen	2	12	(1.9)	43	(3.5)	0.26	–	–	–	–	0.06
	3	552	(89.3)	1064	(86.9)		308	(96.0)	557	(98.1)	
	≥4	54	(8.7)	118	(9.6)		13	(4.1)	11	(1.9)	

ART, antiretroviral therapy; EFV, efavirenz; IQR, interquartile range; NK, not known; NNRTI, nonnucleoside reverse transcriptase inhibitor; NRTI, nucleoside/nucleotide reverse transcriptase inhibitor; PI, protease inhibitor.

^a^Or 9 months prior to pseudo-delivery for controls.

^b^Or in the 8 months prior to pseudo-delivery for controls.

^c^At delivery (postpartum women) or pseudo-delivery (controls).

^d^Characteristics used to identify suitable controls for postpartum women. In addition, postpartum women who started ART during pregnancy were also matched to controls using CD4^+^ cell count at ART start.

^e^Previous live births reported to NSHPC. This does not include live births prior to HIV infection.

**Table 2:** Adjusted hazard ratios for viral rebound in postpartum women conceiving on antiretroviral therapy and controls stratified by time since delivery.

Baseline characteristic at delivery/pseudo-delivery		<3 months since delivery/pseudo-delivery		3–12 months since delivery/pseudo-delivery	
		aHR (95% CI)	*P*	aHR (95% CI)	*P*
Group	Control	Reference	<0.001	Reference	0.76
	Postpartum	2.63 (1.58–4.39)		0.93 (0.59–1.47)	
Calendar year (per additional year)		0.81 (0.70–0.95)	0.01	0.96 (0.84–1.09)	0.50
Age (per 10 additional years)		0.93 (0.55–1.55)	0.77	0.84 (0.55–1.28)	0.42
Ethnicity	Black African	Reference	0.41	Reference	0.59
	White	1.79 (0.87–3.71)		0.75 (0.36–1.57)	
	Black Caribbean	1.52 (0.36–6.35)		0.45 (0.06–3.28)	
	Other/NK	1.34 (0.62–2.91)		0.69 (0.33–1.45)	
Exposure category	Heterosexual sex	Reference	0.63	Reference	0.87
	Injecting drug use	0.41 (0.05–3.72)		0.73 (0.14–3.84)	
	Other/NK	0.56 (0.08–4.17)		1.21 (0.45–3.25)	
Previous live birth		1.31 (0.79–2.20)	0.10	1.37 (0.89–2.11)	0.16
HBV/HCV coinfected		1.43 (0.58–3.55)	0.44	1.25 (0.59–2.65)	0.57
Latest CD4^+^ cell count (cells/μl)	≤200	2.89 (1.14–7.31)	0.10	2.05 (0.96–4.34)	0.05
	201–350	1.74 (0.88–3.46)		1.12 (0.65–1.95)	
	351–500	1.79 (0.96–3.36)		0.64 (0.36–1.13)	
	>500	Reference		Reference	
Duration of ART use	8–12 months	1.34 (0.56–3.25)	0.19	0.98 (0.41–2.34)	0.77
	1–4 years	Reference		Reference	
	≥5 years	0.57 (0.29–1.13)		0.83 (0.50–1.37)	
Time since HIV diagnosis	8–23 months	0.66 (0.25–1.74)	0.69	1.66 (0.82–3.37)	0.04
	2–9 years	Reference		Reference	
	≥10 years	1.04 (0.49–2.21)		1.83 (1.08–3.09)	
Type of ART regimen	PI	1.13 (0.66–1.93)	0.96	1.89 (1.19–3.00)	0.06
	NRTI	–		0.92 (0.12–6.87)	
	NNRTI	Reference		Reference	
	Other	0.95 (0.38–2.34)		1.39 (0.66–2.95)	
Number of drugs in the regimen	2	2.36 (0.51–11.0)	0.17	2.17 (0.73–6.50)	0.01
	3	Reference		Reference	
	≥4	1.86 (0.91–3.81)		2.41 (1.36–4.25)	

Baseline refers to the delivery date (postpartum women) or pseudo-delivery date (controls). aHR, adjusted hazard ratio; ART, antiretroviral therapy; CI, confidence interval; NK, not known; NNRTI, nonnucleoside reverse transcriptase inhibitor; NRTI, nucleoside/nucleotide reverse transcriptase inhibitor; PI, protease inhibitor.

**Table 3:** Adjusted hazard ratios for viral rebound in postpartum women starting antiretroviral therapy during pregnancy and controls stratified by time since delivery.

Baseline characteristic at delivery/pseudo-delivery		<3 months since delivery/pseudo-delivery		3–12 months since delivery/pseudo-delivery	
		aHR (95% CI)	*P*	aHR (95% CI)	*P*
Group	Control	Reference	<0.001	Reference	<0.001
	Postpartum	6.63 (3.58–12.3)		4.05 (2.03–8.09)	
Calendar year (per additional year)		1.02 (0.90–1.16)	0.72	0.83 (0.69–0.99)	0.04
Age (per 10 additional years)		0.71 (0.48–1.05)	0.08	0.51 (0.29–0.90)	0.02
Ethnicity	Black African	Reference	0.81	Reference	0.19
	White	0.68 (0.29–1.61)		1.29 (0.52–3.22)	
	Black Caribbean	1.16 (0.50–2.73)		2.94 (1.11–7.76)	
	Other/NK	0.97 (0.49–1.91)		1.19 (0.52–2.73)	
Exposure category	Heterosexual sex	Reference	0.41	Reference	0.66
	Injecting drug use	4.65 (0.49–44.1)		2.37 (0.25–22.6)	
	Other/NK	1.00 (0.35–2.87)		1.38 (0.47–4.10)	
Previous live birth		1.44 (0.78–2.65)	0.24	1.78 (0.48–6.56)	0.39
HBV/HCV coinfected		0.71 (0.21–2.39)	0.58	1.05 (0.30–3.65)	0.94
Latest CD4^+^ cell count (cells/μl)	≤200	0.18 (0.07–0.48)	<0.001	0.73 (0.32–1.66)	0.35
	201–350	0.39 (0.22–0.70)		0.70 (0.33–1.47)	
	351–500	0.81 (0.49–1.32)		0.44 (0.18–1.08)	
	>500	Reference		Reference	
Duration of ART use	0–2 months	0.82 (0.49–1.38)	0.76	0.91 (0.44–1.87)	0.35
	3–5 months	Reference		Reference	
	6–8 months	–		0.74 (0.26–2.14)	
Time since HIV diagnosis	8–23 months	0.83 (0.51–1.35)	0.73	1.11 (0.55–2.22)	0.62
	2–9 years	Reference		Reference	
	≥10 years	1.08 (0.37–3.11)		0.41 (0.05–3.24)	
Type of ART regimen	PI	0.83 (0.51–1.36)	0.90	1.34 (0.69–2.60)	0.86
	NRTI	–		–	
	NNRTI	Reference		Reference	
	Other	0.89 (0.32–2.52)		–	
Use of EFV-containing regimen		0.20 (0.07–0.60)	0.004	0.88 (0.38–2.06)	0.77
Number of drugs in the regimen	3	Reference	0.86	Reference	0.86
	≥4	1.11 (0.34–3.65)		0.88 (0.20–3.84)	

Baseline refers to the delivery date (postpartum women) or pseudo-delivery date (controls). aHR, adjusted hazard ratio; ART, antiretroviral therapy; CI, confidence interval; EFV, efavirenz; HBV, hepatitis B virus; HCV, hepatitis C virus; NNRTI, nonnucleoside reverse transcriptase inhibitor; NRTI, nucleoside/nucleotide reverse transcriptase inhibitor; PI, protease inhibitor.
